# Dentigerous Cyst and Glandular Odontogenic Cyst: A Rare Combination of Coexisting Pathologies

**DOI:** 10.7759/cureus.76054

**Published:** 2024-12-20

**Authors:** Vasileios Zisis, Nikolaos Shinas, Joe Cordahi, Juliana Robledo, Hassem Geha, Dhurata Shosho, Athanasios Poulopoulos, Deeba Kashtwari

**Affiliations:** 1 Oral Medicine/Pathology, Aristotle University of Thessaloniki, Thessaloniki, GRC; 2 Oral and Maxillofacial Radiology, Henry M. Goldman School of Dental Medicine, Boston, USA; 3 Oral and Maxillofacial Radiology, Comprehensive Dentistry, University of Texas Health Science Center at San Antonio, San Antonio, USA; 4 Pathology and Laboratory Medicine, Long School of Medicine, UT Health, San Antonio, USA; 5 Oral and Maxillofacial Radiology, University of Texas Health Science Center at San Antonio, San Antonio, USA

**Keywords:** cone beam computed tomography (cbct), dentigerous cyst (dc), glandular odontogenic cyst (goc), head and neck pathologies, oral and maxillofacial radiology-related topics, oral biopsy, oral diagnosis and radiology

## Abstract

A dentigerous cyst (DC) is the most common developmental cystic lesion of the jaws. Histologically, these cysts derive from the odontogenic epithelium that includes the reduced enamel epithelium, epithelial cell rests of Serres, and epithelial cell rests of Malassez. Radiographically, DCs are usually presented as well-defined radiolucencies associated with the crown of an unerupted tooth at the level of the cementoenamel junction (CEJ). Glandular odontogenic cysts (GOCs) are classified under the same category as DCs. Radiographically, glandular odontogenic cysts (GOCs) may appear as unilocular or more commonly as multilocular radiolucencies with well-defined margins. It is evident that there is a significant overlap in the radiographic features of the two pathologies. This case report describes one of those cases.

A 49-year-old male patient was referred for a cone beam computed tomography (CBCT) imaging series for the evaluation of possible pathology in areas #17-#19 and ramus to the Graduate Oral and Maxillofacial Radiology Clinic, Health Science Center, San Antonio, University of Texas. The radiographic interpretation revealed a well-defined corticated low-density lesion in the left mandibular molar-ramus region. The mandibular canal was intact and traceable but displaced buccally and inferiorly. The radiographic findings were suggestive of a slow-growing odontogenic process, most likely cystic. Marsupialization and incisional biopsy of the lesion were carried out, which was highly suggestive of GOC. Two months after the initial incisional biopsy, it was decided that enucleation and curettage, as well as extraction of #17, #18, and #19, should be carried out. The enucleated specimen was sent to the histopathology laboratory for evaluation. The second biopsy showed a dentigerous cyst associated with impacted #17. Histopathology continues to be, statistically, the most reliable method for diagnosing these types of abnormalities. However, in certain cases, such as this one, the accuracy of histopathological examination may falter due to overlapping characteristics and different histopathological features based on the location of acquisition of the specimen. The initial radiographic estimation included the differential diagnosis of a DC as a second differential and, although contradicted by the first biopsy result, was eventually supported by the second final biopsy of the entire specimen. Although DCs do not tend to recur, the need for regular follow-ups should not be underestimated, neither by the attending clinician nor by the patients themselves. In conclusion, the radiographically proven, uneventful wound healing constitutes the only reassurance for the patient's well-being.

## Introduction

A dentigerous cyst (DC) is the most common developmental cystic lesion of the jaws with a reported prevalence ranging from 1.44% to 8.64% [1,2]. The classification of this lesion has been standardized and unchanged in the most recent classification by the World Health Organization (WHO) (fifth edition, 2022) [3]. Histologically, these cysts derive from the odontogenic epithelium that includes the reduced enamel epithelium, epithelial cell rests of Serres, and epithelial cell rests of Malassez [4]. Radiographically, DCs are usually presented as well-defined radiolucencies associated with the crown of an unerupted tooth at the level of the cementoenamel junction (CEJ) [3]. Glandular odontogenic cysts (GOCs) are classified under the same category as DCs based on the WHO's classification. Histologically, Kaplan et al. proposed a system of five major and four minor criteria in order to assist and standardize the process of diagnosis [5]. However, due to significant variation in those features, it was proposed that the presence of hobnail cells be the most characteristic microscopic finding [3]. In a later study conducted by Fowler et al., the researchers revised Kaplan's criteria and added one more, the apocrine snouting [6]. The study by Fowler et al. concluded that in order to have a highly predictive diagnosis of GOC, seven or more criteria should be met. On the other hand, five or fewer parameters are highly suggestive of a non-GOC diagnosis [6]. Radiographically, glandular odontogenic cysts (GOCs) may appear as unilocular or more commonly as multilocular radiolucencies with well-defined margins [7]. It is evident that there is a significant overlap in the radiographic features of the two pathologies. In fact, it has been reported in the literature that the overlap of odontogenic pathologies can extend to histological features as well, with evidence of glandular metaplasia [8]. This case report describes one of those cases.

## Case presentation

A 49-year-old male patient was referred for a cone beam computed tomography (CBCT) imaging series for the evaluation of possible pathology in areas #17-#19 and ramus to the Graduate Oral and Maxillofacial Radiology Clinic, Health Science Center, San Antonio, University of Texas.

The patient was asymptomatic and did not report any pain, swelling, fever, chill, nausea, vomiting, and/or dysphagia. A CBCT examination with the Planmeca ProMax3D (Helsinki, Finland) was performed, and an 8 cm × 5 cm volume was acquired.

**Figure 1:**
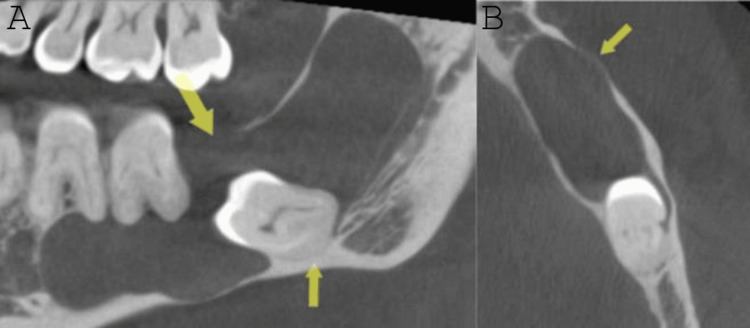
A: The yellow arrows show the unilocular, cystic-like, intrabony lesion. B: The loss of cortical integrity can be noticed.

The radiographic interpretation revealed a well-defined corticated low-density lesion in the left mandibular molar-ramus region. Mesio-distally, it extended from the level of the mesial root of tooth #19 to the mid-height of the ramus. Superio-inferiorly, it extended from the alveolar crest and the anterior part of the ramus to the inferior border of the mandible and the superior border of the inferior alveolar canal. Bucco-lingually, it covered the width of the mandible. There was evidence of interruption of the alveolar crest, thinning of the inferior, buccal, and lingual cortical outlines of the mandible, and impaction of #17 with evidence of attachment of the lesion at the level of the cementoenamel junction (CEJ), more prominently on the mesial aspect, and inferior displacement. No root resorption was noted (Figure 1).

**Figure 2:**
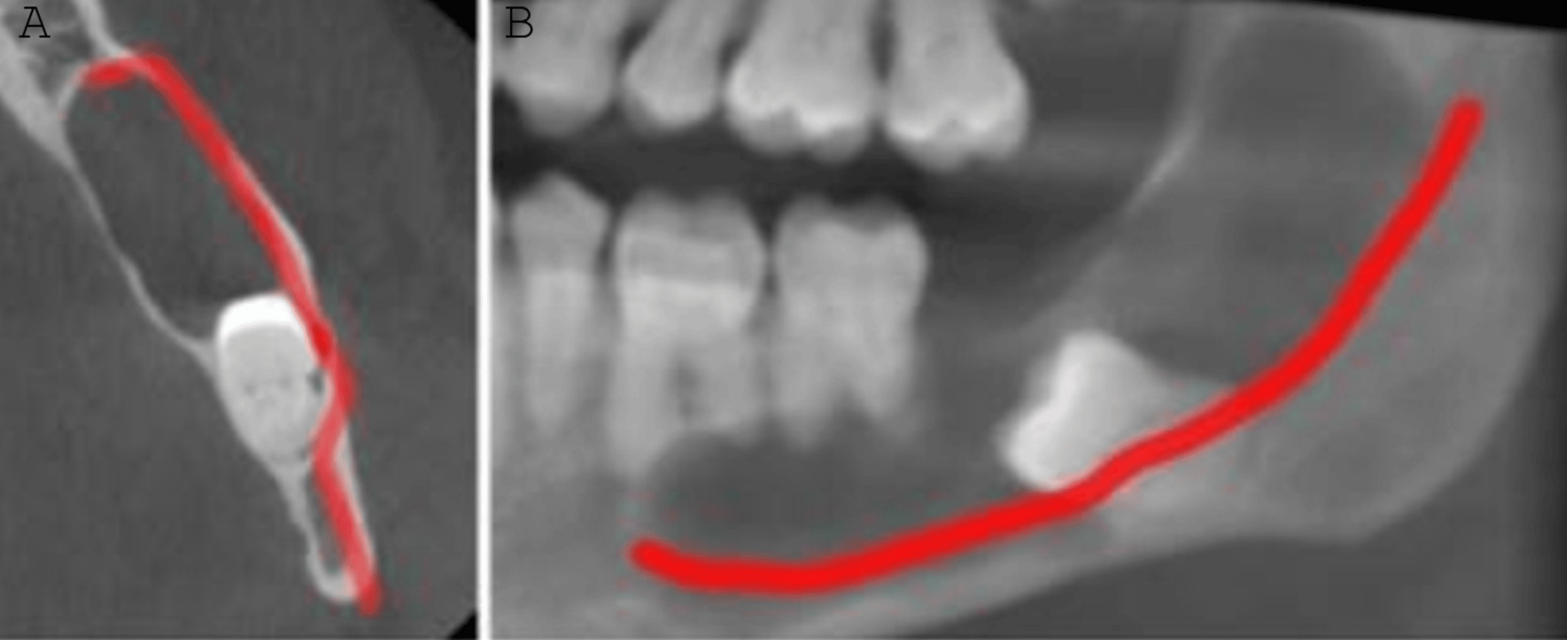
The red line shows the location of the mandibular canal and its close proximity to the impacted tooth (A: transverse plane, B: coronal plane).

The mandibular canal was intact and traceable but displaced buccally and inferiorly (Figure 2).

The radiographic findings were suggestive of a slow-growing odontogenic process, most likely cystic. The most likely diagnosis was an odontogenic keratocyst (OKC) (the antero-posterior dimension is greater than the bucco-lingual dimension with involvement of the peri-coronal sac of impacted #17), followed by dentigerous cyst considering the favorable relation with the cementoenamel junction and unicystic ameloblastoma, as a more unlikely diagnosis. Hence, a biopsy was advised to reach a definitive diagnosis and rule out the other possibilities.

**Figure 3:**
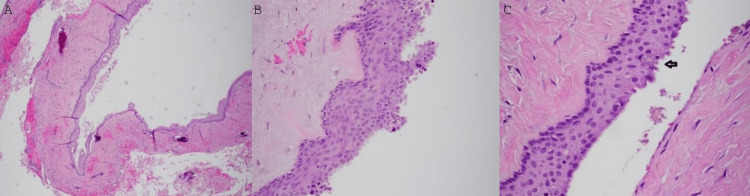
H&E (magnification ×4 (A), ×20 (B), and ×40 (C)): Incisional biopsy specimen showing cystic lining with squamous epithelium. The epithelial cells appear cuboidal to columnar in some areas with a somewhat pebbly to papillary surface and a ciliated surface in other areas. There are scattered mucus cells throughout the cystic lining. In focal areas of the lining, there are spherical nodules of epithelium. Multiple duct-like or microcystic structures are seen throughout the squamous lining. The fibrous connective tissue wall is dense and well-vascularized. There are scattered lymphocytes throughout the fibrous wall. H&E: hematoxylin and eosin

Marsupialization (to avoid if possible, nerve damage) and incisional biopsy of the lesion were carried out. During the histopathological examination, the biopsy specimen was reported to be a cyst lined by squamous epithelium. The epithelial cells appeared cuboidal to columnar in some areas with a somewhat pebbly to papillary surface and a ciliated surface in other areas. There were scattered mucus cells throughout the cystic lining. In focal areas of the lining, there were spherical nodules of epithelium. Also, multiple duct-like or microcystic structures were seen throughout the squamous lining. The fibrous connective tissue wall was dense and well-vascularized. Scattered lymphocytes were detected throughout the fibrous wall. Findings were highly suggestive of a GOC (Figure 3).

Two months after the initial incisional biopsy, it was decided that enucleation and curettage, as well as extraction of #17, #18, and #19, should be carried out. The enucleated specimen was sent to the histopathology laboratory for evaluation.

**Figure 4:**
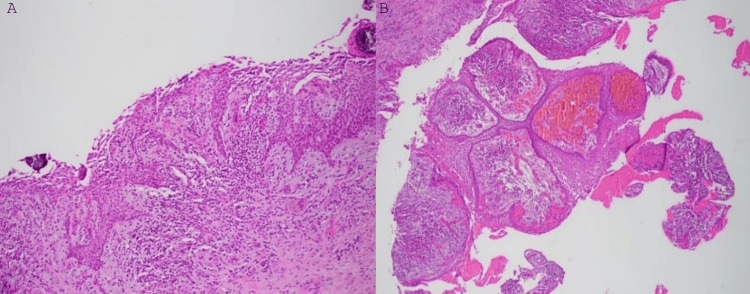
H&E (magnification ×10 (A and B)): Second biopsy specimen showing a cyst. The lumen was lined by hyperplastic and edematous stratified squamous epithelium. The underlying fibrous connective tissue wall was composed of loosely and densely arranged collagen fibers with areas of myxoid change. A dense inflammatory infiltrate composed of lymphocytes and plasma cells was present throughout the cyst wall. H&E: hematoxylin and eosin

After examining the specimen, histopathology confirmed that the specimen was cystic and the lumen was lined by hyperplastic and edematous stratified squamous epithelium. The underlying fibrous connective tissue wall was composed of loosely and densely arranged collagen fibers with areas of myxoid change. A dense inflammatory infiltrate composed of lymphocytes and plasma cells was present throughout the cyst wall. The second biopsy showed a dentigerous cyst associated with impacted #17 (Figure 4).

**Figure 5:**
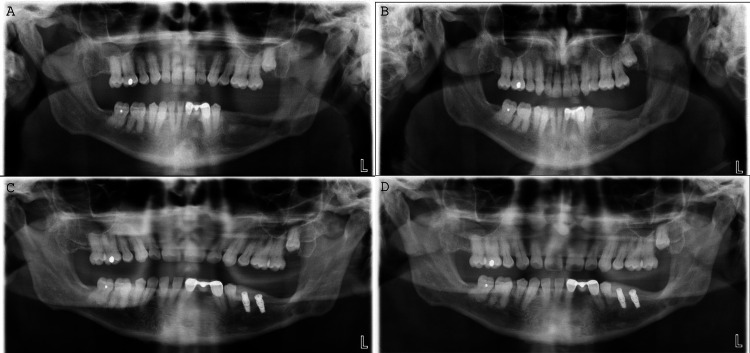
A: OPG examination three months post-surgery. B: OPG examination nine months post-surgery. C: OPG examination two years and two months post-surgery. The edentulous area was eventually rehabilitated through implants. D: OPG examination two years and seven months post-surgery. OPG: orthopantomogram

The following orthopantomogram (OPG) examinations illustrate the gradual healing of the lesion three months (Figure 5A), nine months (Figure 5B), two years and two months (Figure 5C), and two years and seven months (Figure 5D) post-surgery.

## Discussion

The overlapping features of different types of cysts render proper diagnosis and treatment crucial. From a clinical and radiographic perspective, the lesions closely resemble one another, leading to a challenging diagnostic situation for clinicians. Histopathology continues to be, statistically, the most reliable method for diagnosing these types of abnormalities. However, in certain cases, such as this one, the accuracy of histopathological examination may falter due to overlapping characteristics and different histopathological features based on the location of acquisition of the specimen. The presence of multipotent cells within the cystic lumen of odontogenic cysts leads to variances and diversities [6]. There is a case report in the literature where an odontogenic keratocyst (OKC) was observed with concurrent glandular epithelium features indicating the synchronous presence of GOC [6]. A similar scenario may be safely assumed for our case as well.

DCs account for approximately 24% of all true cysts in the jaws [9], and the typical age distribution is the second and third decades of life [3]. Radiographically, these lesions are associated with an impacted tooth at the level of the CEJ [3] and are well-defined and unilocular [9].

On the other hand, GOC constitutes an uncommon cyst of odontogenic origin, and its prevalence varies from 0.012% to 1.3% of all jaw cysts, with the mean being 0.17% [10]. The most common location for GOC was identified to be the anterior mandible. However, in approximately 14%-24% of cases, GOC may grow to a large size, resulting in jaw swelling and causing paresthesia and numbness in the affected area [11,12]. The condition may affect individuals across a broad range of ages; however, it is most commonly identified in patients between their 50s and 70s with a slight male predilection [13]. GOC often appears radiographically as a cystic lesion, which can be either unilocular or multilocular [12]. The lesion has well-defined boundaries, although there may be instances when the cortical integrity is compromised, cortices expand, and, in some cases, root resorption occurs [12,14]. In certain cases, GOC is linked with unerupted teeth. The histological diagnosis of GOC can be challenging due to the rarity of this clinical entity and the presence of both benign and malignant lesions in the differential diagnosis. In particular, the differential diagnosis includes radicular cysts, dentigerous cysts with or without metaplastic changes, certain types of ameloblastoma, and more severe conditions such as low-grade mucoepidermoid carcinoma (MEC) [5,6,15].

The fact that the patient did not manifest any symptoms or clinical signs may be deemed as atypical. Normally, swelling is the most common clinical sign, followed by pain, and sometimes numbness [12]. GOC's recurrence rate is higher when choosing a conservative surgical treatment [5]. Additionally, larger lesions have a higher recurrence rate [16]. However, in our case, the decision was made for enucleation rather than surgical resection to avoid the detrimental effect that the surgical resection would have on the patient's quality of life. The case has been followed up for two years and seven months so far, and there is no indication of recurrence. The patient should be kept under follow-up for at least three years [13].

## Conclusions

Although excisional biopsy is the golden standard by which we distinguish such lesions, it should not be taken solely and indiscriminately into account. Such extreme cases highlight the crucial role played by imaging that can significantly aid in arriving at a proper diagnosis. The initial radiographic estimation included the differential diagnosis of a DC as a second differential and, although contradicted by the first biopsy result, was eventually supported by the second final biopsy of the entire specimen. Although DCs do not tend to recur, the need for regular follow-ups should not be underestimated, neither by the attending clinician nor by the patients themselves. In conclusion, the radiographically proven, uneventful wound healing constitutes the only reassurance for the patient's well-being.

## References

[1] Aldelaimi AA, Enezei HH, Berum HE, Abdulkaream SM, Mohammed KA, Aldelaimi TN (2024). Management of a dentigerous cyst; a ten-year clinicopathological study. BMC Oral Health.

[2] Huang G, Moore L, Logan RM, Gue S (2019). Histological analysis of 41 dentigerous cysts in a paediatric population. J Oral Pathol Med.

[3] Soluk-Tekkesin M, Wright JM (2022). The World Health Organization classification of odontogenic lesions: a summary of the changes of the 2022 (5th) edition. Turk Patoloji Derg.

[4] Malas V, Rasmusson L (2024). Odontogenic cysts: presentation of a simplified classification system. Clin Surg.

[5] Kaplan I, Anavi Y, Hirshberg A (2008). Glandular odontogenic cyst: a challenge in diagnosis and treatment. Oral Dis.

[6] Fowler CB, Brannon RB, Kessler HP, Castle JT, Kahn MA (2011). Glandular odontogenic cyst: analysis of 46 cases with special emphasis on microscopic criteria for diagnosis. Head Neck Pathol.

[7] Shah M, Kale H, Ranginwala A, Patel G (2014). Glandular odontogenic cyst: a rare entity. J Oral Maxillofac Pathol.

[8] Lohokare AU, Nisa SU, Mhapuskar A, Hiremutt DR, Thopte S (2022). Odontogenic keratocyst with diverse variations: a rare case report. Ann Maxillofac Surg.

[9] Aher V, Chander PM, Chikkalingaiah RG, Ali FM (2013). Dentigerous cysts in four quadrants: a rare and first reported case. J Surg Tech Case Rep.

[10] Gurler G, Al-Ghamian H, Aksakalli N, Delilbasi C (2017). Glandular odontogenic cyst: case series. Contemp Clin Dent.

[11] Chrcanovic BR, Gomez RS (2018). Glandular odontogenic cyst: an updated analysis of 169 cases reported in the literature. Oral Dis.

[12] Ferreira JC, Vêncio EF, de Sá RT, Gasperini G (2019). Glandular odontogenic cyst in dentigerous relationship: an uncommon case report. Case Rep Dent.

[13] Kochaji N, Alhessani S, Ibrahim S, Al-Awad A (2023). Posterior mandibular glandular cyst: a rare case report. Int J Surg Case Rep.

[14] Tambawala SS, Karjodkar FR, Yadav A, Sansare K, Sontakke S (2014). Glandular odontogenic cyst: a case report. Imaging Sci Dent.

[15] Dunfee BL, Sakai O, Pistey R, Gohel A (2006). Radiologic and pathologic characteristics of benign and malignant lesions of the mandible. Radiographics.

[16] Poudel P, Srii R, Chaurasia N, Upadhyaya C (2020). Glandular odontogenic cyst-report of a rare case. Clin Case Rep.

